# Detection of Lung Cancer Biomarkers C_3_H_6_O, CH_2_O, and C_5_H_8_ through Pd‑, Pt‑, and Ag-Doped BC_6_N Monolayers: A Density Functional Theory Study

**DOI:** 10.1021/acsomega.5c07373

**Published:** 2025-10-24

**Authors:** Charlotte Lai, Xuan Luo

**Affiliations:** 528218 National Graphene Research and Development Center, Springfield, Virginia 22151, United States

## Abstract

Early detection of lung cancer remains a major clinical challenge, limited by the high cost, invasiveness, and insufficient sensitivity of current diagnostic methods. Exhaled volatile organic compounds (VOCs), such as acetone (C_3_H_6_O), formaldehyde (CH_2_O), and isoprene (C_5_H_8_), have emerged as promising noninvasive biomarkers, with elevated concentrations observed in the breath of lung cancer patients. In this study, we employ density functional theory (DFT) to evaluate the sensing performance of pristine and transition-metal (TM)-doped (Pd, Pt, and Ag) BC_6_N monolayers toward these VOCs. TM doping was found to significantly enhance adsorption strength and sensing response compared to the pristine surface, with Pd-, Pt-, and Ag-doped BC_6_N exhibiting notably greater adsorption energies (−0.295 to −2.211 eV) than the pure monolayer (0.001 to −0.086 eV). Among the dopants, Ag–BC_6_N displays the most favorable sensing properties, including moderate adsorption energies (−0.295 to −0.535 eV), short adsorption distances (2.23–2.31 Å), and rapid recovery times (9.74 × 10^–4^ to 9.84 × 10^–8^ s), indicating superior reversibility relative to Pd and Pt. Band structure, charge transfer, and projected density of states (PDOS) analyses further reveal strong orbital hybridization and electronic modulation upon VOC adsorption. These findings establish Ag–BC_6_N, in particular, as a highly promising sensor for sensitive, scalable, and reusable detection of lung cancer biomarkers via breath analysis.

## Graphical abstract

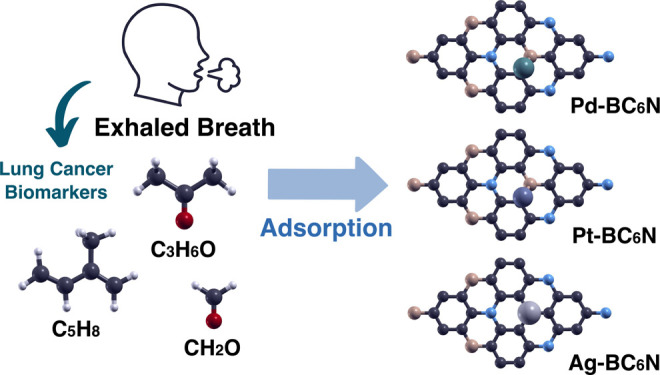

## Introduction

Lung cancer remains the leading cause of cancer-related deaths worldwide, accounting for 18% of the global cancer burden and exhibiting disproportionately high mortality rates.^1,2^ In the United States, the disease was responsible for an estimated 125,070 deaths in 2024—the highest among all cancer types.^3^ Despite significant advances in diagnosis and treatment, the five-year survival rate remains critically low, underscoring the need for early detection to improve patient outcomes.^4,5^ Historically, screening relied on chest X-rays and sputum cytology, but these methods demonstrated limited sensitivity and failed to reduce overall mortality.^6–8^ Newer technologies like proteomic profiling,^9^ nuclear magnetic resonance (NMR) spectroscopy,^10^ and computed tomograpy (CT)^11^ also remain impractical for large-scale screening due to high costs, long processing times, and risks of overdiagnosis.^12–14^ These limitations, coupled with the strong association between late-stage detection and poor prognosis, highlight the need for a sensitive, cost-effective, and scalable diagnostic solution.^5,15^

In recent years, exhaled breath analysis has emerged as a promising noninvasive diagnostic tool, leveraging disease-specific volatile organic compounds (VOCs) as biomarkers.^16^ The human breath contains over 1400 VOCs, many of which correlate with physiological and pathological conditions.^17^ For example, elevated levels of heptanal have been associated with COVID-19,^18,19^ while cyclohexane and benzene derivatives are linked to tuberculosis (TB).^20^ In the context of lung cancer, VOCs such as acetone (C_3_H_6_O),^21^ formaldehyde (CH_2_O),^22^ and isoprene (C_5_H_8_)^23^ have been identified as key biomarkers.

For this study, these three molecules were specifically selected for their well-established role in lung cancer pathology, prevalence in clinical studies, and relatively high concentrations, which facilitate detection.^24^ Their clinical relevance is demonstrated by significant concentration shifts in the exhaled breath of lung cancer patients, with acetone (>1000 ppb), formaldehyde (≈83 ppb), and isoprene (≈112 ppb) deviating markedly from healthy baselines (300–900 ppb, ≈48 ppb, and ≈221 ppb, respectively).^21,22,25–27^ Among them, formaldehyde is particularly critical both as a Class I human carcinogen and due to its endogenous production in lung cancer cells.^28,29^ Nevertheless, as these VOCs are not unique to lung cancer and can be characteristic of other diseases (e.g., acetone for diabetes,^30^ isoprene for advanced liver fibrosis^31^), the diagnostic power relies on the codetection of these three molecules via multivariate analysis to establish a discriminatory breathprint.^32^

For this approach to be clinically viable, a sensing platform must offer both high analytical precision and practical utility. Currently, Gas Chromatography–Mass Spectrometry (GC–MS) remains the gold standard for breath-based VOC detection, offering high precision and the ability to identify a wide range of compounds.^33^ However, its reliance on costly instrumentation, complex sample preparation, and the need for specialized personnel limits widespread clinical adoption.^34,35^ In contrast, room-temperature, nanomaterial-based gas sensors have emerged as compelling alternatives due to advantages such as high sensitivity, rapid response times, and low operational costs.^36,37^ Among these, carbon nanotubes (CNTs) have demonstrated exceptional performance due to their high specific surface area and excellent electrical conductivity, enabling efficient VOC adsorption.^38^ Comparatively, metal oxide semiconductors (MOS) such SnO_2_ and ZnO have shown remarkable sensitivity in detecting lung cancer biomarkers such as acetone and formaldehyde.^39,40^ More recently, two-dimensional (2D) materials have garnered attention due to their atomically thin structures, large surface-to-volume ratios, and high surface activities, which make them particularly well-suited for capturing molecular interactions at trace levels.^41,42^ Various 2D materials have been investigated for lung cancer-related biomarker detection. For instance, recent advances in hexagonal boron nitride (*h*-BN) have shown potential in detecting VOCs such as acetone, benzene, and isoprene, with Sn doping vastly improving interaction strength and adsorption energy.^43,44^ Similarly, transition metal dichalcogenides (TMDs), including MoS_2_ and WS_2_ demonstrate promise in diabetes-related acetone sensing as well as in detecting cancer-related VOCs like isoprene.^45,46^ Kumar et al. and Panigrahi et al. have further shown the exceptional adsorption strength and favorable electronic properties of Ti_3_C_2_T_*x*_ MXenes toward a range of lung cancer biomarkers.^47,48^

Among emerging 2D materials, graphene stands out as one of the most prominent and transformative.^19,49^ Its exceptional properties—including excellent chemical stability, mechanical strength, and tunable electronic structure—have made it a prime candidate for biosensing applications.^50^ However, pristine graphene’s zero band gap limits its sensitivity and selectivity toward diverse VOCs, prompting investigations in doping, functionalization, and defect engineering to enhance its performance.^51,52^ In particular, substitutional doping with boron (B) and nitrogen (N) have proven effective in opening graphene’s band gap and improving its adsorption capability for gas molecules.^53,54^ Because B and N have atomic radii similar to carbon (C), they can be substitutionally incorporated into the graphene lattice without much distortion, giving rise to graphene-like boron carbide (BC_3_) and carbon nitride (C_3_N).^55,56^ For instance, Zhao et al. demonstrated that BC_3_ exhibits high sensitivity and selectivity toward gases such as CH_3_COCH_3_, H_2_, CO_2_, and SO_2_, specifically highlighting its promise for acetone detection.^57^ Beheshtian et al. further enhanced BC_3_’s sensing capabilities by doping it with Al and Si, resulting in stronger adsorption interactions with formaldehyde.^58^ Moreover, Azam et al. investigated the electrochemical sensing potential of C_3_N for hydrogen-containing toxic analytes,^59^ while Ma et al. found that pristine C_3_N serves as an effective room-temperature sensor for NO_2_, with enhanced performance achieved through boron doping.^60^

Following these developments, borocarbonitride BC_6_N—a transitional structure between BC_3_ and C_3_N—was recently synthesized via a two-step borylation reaction.^61^ While structurally similar to graphene, BC_6_N possesses a direct band gap of approximately 1.27 eV and high carrier mobility comparable to that of black phosphorene, enabling semiconducting behavior ideal for electronic and sensing applications.^62,63^ This tunability arises from the acceptor properties of boron and donor properties of nitrogen, while its high carbon content helps retain graphene-like characteristics such as thermal conductivity and mechanical strength.^64,65^ Baachaoui et al. demonstrated that covalently functionalized BC_6_N can selectively detect NH_3_ due to the presence of hydrogen bonding interactions.^66^ In recent years, several studies have also investigated the gas-sensing capabilities of transition metal (TM)-doped BC_6_N. For example, Aasi et al. reported that Pd doping heightens the monolayer’s sensitivity to NO_*x*_ gases,^64^ while Alghamdi et al. found that Pt doping leads to improved adsorption performance toward NH_3_, NO, and NO_2_.^67^ Similarly, Jiang and Luo demonstrated that Pd doping enhances its adsorption energy and charge transfer toward acetone, benzene, and tetrachloromethane.^62^ Aghaei et al. further showed that introducing a single carbon vacancy in the monolayer increases adsorption capacity for VOCs such as acetone, ethanol, methanol, formaldehyde, and toluene.^68^

Based on these findings, we investigated the potential of BC_6_N monolayers for detecting VOCs related to lung cancer. This material was selected as a promising yet less-explored platform compared to the more extensively studied 2D materials like TMDs or MXenes, offering a unique combination of a tunable band gap, high carrier mobility, and a heterogeneous surface chemistry ideal for VOC capture. Using first-principles calculations based on density functional theory (DFT),^69^ we examined the interactions between BC_6_N and acetone, formaldehyde, and isoprene—representative biomarkers which correlate to ketone, aldehyde, and hydrocarbon functional groups, respectively. To improve the monolayer’s chemical reactivity and sensing performance, we introduced Pd, Pt, and Ag dopants into the BC_6_N lattice and analyzed the structural and electronic behavior of each system. Dopant selection was based on their established efficacy in similar sensor materials: Pd and Pt show superior response to oxygenated VOCs, while Ag is a cost-effective alternative showing adequate adsorption performance for hydrocarbons and aldehydes.^19,64,67^ Au was excluded as a dopant, as it has shown relatively weaker reversibility and sensing performance with these specific VOCs, yielding lower sensitivity suboptimal for trace detection in comparative sensor materials.^70,71^ Although prior studies have demonstrated TM-doped BC_6_N’s effectiveness for general gas detection, research into its viability for sensing lung cancer-specific biomarkers remain limited. Through this theoretical framework, we aim to compare and evaluate the adsorption performances of the pristine and doped BC_6_N monolayers to determine an effective sensor for the three VOCs.

## Methods

### Computational Details

All calculations were performed using density functional theory (DFT)^69^ as implemented in the ABINIT package.^72^ The exchange–correlation interactions were treated using the generalized gradient approximation (GGA)^73^ in the Perdew–Burke–Erzerhof (PBE) form. Pseudopotentials were generated using the projector augmented wave (PAW)^74^ method via the AtomPAW code,^75^ with the electron configurations and corresponding cutoff radii summarized in Table 1.

**Table 1:** Electron Configurations and Radius Cutoffs of Elements Used to Generate PAW Pseudopotentials

element	electron configuration	radius cutoffs (Bohr)
hydrogen (H)	1s^1^	0.99
boron (B)	[He]2s^2^2p^1^	1.70
carbon (C)	[He]2s^2^2p^2^	1.51
nitrogen (N)	[He]2s^2^2p^3^	1.20
oxygen (O)	[He]2s^2^2p^4^	1.41
palladium (Pd)	[Ar 3d^10^]4s^2^4p^6^5s^1^4d^9^	2.51
platinum (Pt)	[Xe 4f^14^]6s^1^5d^9^	2.50
silver (Ag)	[Kr]5s^1^4d^10^	2.50

**Table 2:** Optimized Structural and Electronic Properties of the Pristine and Doped BC_6_N Monolayers, including Lattice Constant (*a*), Bond Lengths between B–C, C–C, C–N, TM–B, and TM–C Atom Pairs (*d*), Defect Formation Energy (*E*_form_), and Energy Band Gap (*E*_g_)

system	*a* (Å)	*d*_B–C_ (Å)	*d*_C–C_ (Å)	*d*_C–N_ (Å)	*d*_TM–B_ (Å)	*d*_TM–C_ (Å)	*E*_form_ (eV)	*E*_g_ (eV)
pristine BC_6_N	4.975	1.47	1.41	1.45	N/A	N/A	N/A	1.273
Pd–BC_6_N	4.981	1.48	1.43	1.45	2.24	2.13	2.277	1.069
Pt–BC_6_N	4.979	1.49	1.44	1.44	2.20	2.06	3.570	0.870
Ag–BC_6_N	4.976	1.49	1.41	1.46	2.38	N/A	2.436	0.543

Dispersion-corrected (van der Waals) functionals were not employed in the primary calculations. This choice was made after comparative tests indicated that while such corrections increase the absolute adsorption energies by approximately an order of magnitude, the critical relative trends and energy differences between the studied systems—the focus of this comparative screening study—remain consistent. We strive to incorporate these corrections in future work for quantitative precision.

Self-consistent field (SCF) iterations for total energy calculations were considered converged when the energy difference between successive steps was less than 1.0 × 10^–10^ Ha. To ensure numerical accuracy, convergence tests were then performed for the kinetic energy cutoff, Monkhorst–Pack *k*-point grids, and vacuum layer. These parameters were considered converged when the total energy variation between two consecutive data sets fell below 0.0001 Ha (≈0.003 eV), a threshold commonly used in first-principles studies.^62,70^ The final calculations employed a plane-wave kinetic energy cutoff of 25 Ha (∼680 eV). A *k*-point mesh converged to 4 × 4 × 1 was used for the primitive (1 × 1) BC_6_N unit cell. For the larger 2 × 2 supercells used in this study, a 2 × 2 × 1 grid was employed to maintain a comparable sampling density in the Brillouin zone during structural relaxations.^76^

To mitigate the cell size effect, we converged the unit cell according to the convergence criterion of 0.0001 (≈3 meV), as described previously. The converged value for acetone, formaldehyde, and isoprene are 21, 14, and 20 Bohr, respectively. The distance between each molecule and their images are thus 21, 14, and 20 Bohr, respectively. Considering this, we selected the monolayer size and vacuum height accordingly, such that a 2 × 2 supercell was used and a vacuum layer of 20 Å was applied in the nonperiodic (*z*) direction to prevent interactions between periodic images. This sufficiently large, converged unit cell ensures that the calculation of adsorption energies and other properties models a single molecule on an infinite sheet.

Subsequently, structural optimizations were carried out for the three VOC molecules, the pristine and TM-doped BC_6_N monolayers, and their corresponding adsorption complexes. These calculations employed the Broyden–Fletcher–Goldfarb–Shanno (BFGS) minimization algorithm,^77^ with a maximum force tolerance of 2.0 × 10^–4^ Ha/bohr (0.01 eV/Å) applied to each atom. To validate the thermal stability of the pristine and TM-doped BC_6_N monolayers, molecular dynamics (MD) simulations were conducted at 300 K. The MD process involved an iterative cycle: after each molecular dynamics step, a self-consistent field (SCF) calculation was performed. This cycle was repeated until the convergence criterion was met, which was defined as the maximum force on all atoms being smaller than the force tolerance of 2.0 × 10^–4^ Ha/bohr (0.01 eV/Å).

### Atomic Structures

Molecular structures of acetone (C_3_H_6_O), formaldehyde (CH_2_O), and isoprene (C_5_H_8_) were sourced from PubChem and subsequently subject to structural optimization, as shown in Figure 1. The structural and electronic properties of both pristine and TM-doped BC_6_N monolayers were then investigated. We began with the optimization of a 1 × 1 primitive BC_6_N cell, which exhibits a graphene-like lattice in which two carbon atoms are substitutionally doped with boron and nitrogen. To enable detailed adsorption analysis, the optimized unit cell was expanded into a 2 × 2 supercell consisting of 32 atoms (Figure 2a). TM doping was introduced by interstitially incorporating a single Pd, Pt, or Ag atom at one of seven candidate sites, as labeled in Figure 2a. Finally, each optimized VOC molecule was positioned above the monolayer surface, and full structural optimizations were performed to evaluate the resulting adsorption configurations and interactions.

**Figure 1:**
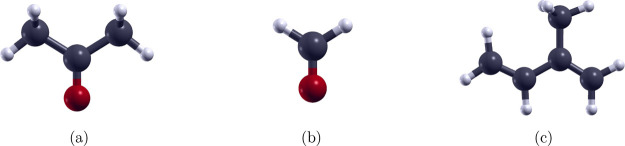
Optimized atomic structures of (a) acetone (C_3_H_6_O), (b) formaldehyde (CH_2_O), and (c) isoprene (C_5_H_8_). White, dark gray, and red colors represent C, H, and O atoms, respectively.

**Figure 2:**
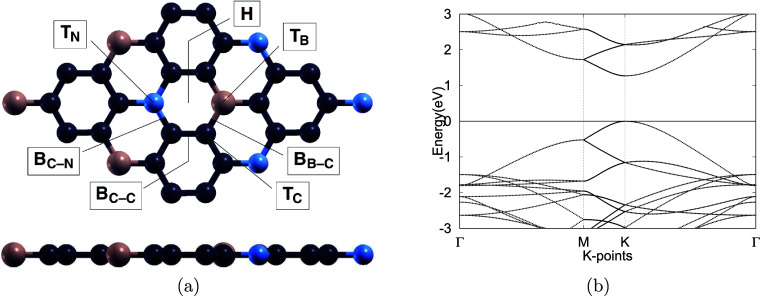
(a) Top and side views of the optimized 2 × 2 BC_6_N monolayer. Seven potential doping sites for Pd, Pt, and Ag atoms are indicated: the hollow site (*H*), bridge sites above B–C, C–C, and C–N bonds (*B*_B–C_, *B*_C–C_, and *B*_C–N_), and top sites above B, C, and N atoms (*T*_B_, *T*_C_, and *T*_N_). B, C, and N atoms are shown in pink, dark gray, and blue, respectively. (b) Electronic band structure of the pristine BC_6_N monolayer, with the Fermi level set to zero.

### Energy Calculations

#### Defect Formation Energy

The defect formation energy (*E*_form_)^19,70^ of the Pd-, Pt-, and Ag-doped BC_6_N monolayers was computed using

$$E_{\mathrm{form}}=E_{\mathrm{TM}-\mathrm{monolayer}}-E_{\mathrm{monolayer}}-E_{\mathrm{TM}}$$ 1

where *E*_TM–monolayer_, *E*_monolayer_, and *E*_TM_ represent the total energy of the doped BC_6_N monolayer, pristine BC_6_N monolayer, and the chemical potential of the Pd, Pt, or Ag atom, respectively.

#### Adsorption Energy

Total energy calculations were performed for the optimized VOC molecules, pristine and TM-doped BC_6_N monolayers, and VOC–BC_6_N complexes. The adsorption energy (*E*_ad_)^57,59,78^ for each system was calculated by

$$E_{\mathrm{ad}}=E_{\mathrm{VOC}-\mathrm{monolayer}}-E_{\mathrm{monolayer}}-E_{\mathrm{VOC}}$$ 2

in which *E*_VOC–monolayer_, *E*_monolayer_, and *E*_VOC_ represent the total energy of the VOC–BC_6_N configurations, the BC_6_N monolayer, and the VOC molecule, respectively.

### Electronic Structure

#### Band Structure

Band structure calculations were performed for the pristine and transition-metal-doped monolayers, as well as their corresponding VOC adsorption complexes, using the optimized geometries of acetone, formaldehyde, and isoprene adsorbed on each monolayer. These calculations were based on the converged charge densities obtained from prior structural relaxations. The band structures were computed along the high-symmetry *k*-points $\Gamma (0,0,0),M\left(\frac{1}{2},\frac{1}{2},0\right),K\left(\frac{2}{3},\frac{1}{3},0\right)$ , and returning to Γ.

To quantify changes in the band structures, the percentage change in the band gap (Δ*E*_g_)^62^ was calculated using

$$\Delta E_{g}=\frac{E_{g}2-E_{g}1}{E_{g}1}\times 100\%$$ 3

in which *E*_g_1 is the band gap of the monolayer before VOC adsorption, and *E*_g_2 is the band gap after adsorption.

#### Projected Density of States

The projected density of states (PDOS) was computed using the tetrahedron method to further analyze the VOC–BC_6_N adsorption systems. The atoms situated nearest to the gas adsorption site were chosen for projection. Thus, the PDOS of the 2p orbital of oxygen in acetone and formaldehyde, the 2p orbital of carbon in isoprene, the 2p orbital of boron in BC_6_N, and the d orbital of the TM dopants were plotted for VOC-adsorbed BC_6_N systems.

#### Charge Transfer

To further investigate the interaction between VOC molecules and the BC_6_N monolayer, we calculated and analyzed the charge transfer,^79–81^ as given by

$$\Delta \rho =\rho_{\mathrm{VOC}-\mathrm{monolayer}}-\rho_{\mathrm{monolayer}}-\rho_{\mathrm{VOC}}$$ 4

where Δρ represents the net charge transfer, ρ_VOC–monolayer_ is the total charge density of the VOC–BC_6_N complex, and ρ_monolayer_ and ρ_VOC_ are the charge densities of the isolated monolayer and VOC molecule, respectively. Charge redistribution upon adsorption was qualitatively assessed by visualizing the regions of electron accumulation and depletion in the difference charge density plots.

### Recovery Time, Conductivity, and Sensitivity

The reusability of the sensing material was assessed by calculating the recovery times of pristine and doped BC_6_N monolayers following VOC adsorption. According to the conventional transition state theory,^19^ the recovery time (τ),^64,68^ in seconds, can be described by the following relation:

$$\tau =\nu^{-1}\exp\left(-\frac{E_{\mathrm{ad}}}{k_{B}T}\right)$$ 5

Here, ν represents the attempt frequency (ν = 10^12^ s^–1^ for visible light), *k*_B_ represents the Boltzmann constant (*k*_B_ = 8.617 × 10^–5^ eV K^–1^), and *T* represents the thermodynamic temperature.^70,71,80^ All calculations in this work were performed at *T* = 300 K to establish a room-temperature performance benchmark.

It is important to note that recovery time exhibits an exponential dependence on operating temperature.^82,83^ In practical sensor applications, elevated temperatures are often employed to significantly shorten response time and enhance reversibility,^84^ albeit with an associated increase in power consumption;^85^ this remains a vital parameter for optimization in future operational designs.

The electrical conductivity (σ)^70,86^ of the system is given by

$$\sigma \propto \exp\left(-\frac{E_{g}}{2k_{B}T}\right)$$ 6

where *E*_g_ is the band gap of the BC_6_N system, *k*_B_ is the Boltzmann constant, and *T* is the thermodynamic temperature.

This semiclassical approach is a widely adopted metric in DFT-based sensing studies for evaluating conductivity changes upon gas adsorption; it provides a reliable qualitative measure of the sensing response at 300 K, where a reduced band gap correlates with heightened conductivity.^68,86^ While quantum-mechanical methods like NEGF-DFT offer quantitative precision for nanoscale transport, the present approach is well-suited for fundamental screening.^64^ A necessary extension of this work should involve employing these advanced quantum transport techniques to better model device-level performance.

Furthermore, we analyzed the sensitivity of the monolayers, an important factor for evaluating sensor performance. Sensitivity (*S*)^19,87^ was defined as the variation of the electrical conductivity of the BC6N monolayer before and after VOC adsorption:

$$S=\left|\frac{\sigma_{\mathrm{VOC}+\mathrm{monolayer}}-\sigma_{\mathrm{monolayer}}}{\sigma_{\mathrm{monolayer}}}\right|\times 100\%$$ 7

Specifically, σ_monolayer_ and σ_VOC+monolayer_ denote the conductivities of the pristine or TM-doped BC_6_N monolayer and the VOC–BC_6_N complex, respectively. A high positive sensitivity value indicates a substantial enhancement of conductivity upon gas exposure, confirming a strong sensing response.

## Results and Discussion

We first examined the adsorption behavior of pristine BC_6_N toward acetone, formaldehyde, and isoprene, revealing weak interactions with the VOC molecules. To improve adsorption strength and electronic responsiveness, Pd, Pt, and Ag were introduced as interstitial dopants. It is important to highlight that the electronic impact of interstitial doping is distinct from substitutional doping. Because these dopants were incorporated into interstitial sites within the BC_6_N lattice rather than replacing host atoms, they do not create the charge states responsible for traditional n-type or p-type behavior. Therefore, the appearance of the Fermi level near the valence band in Figure 3 should not be interpreted as evidence of p-type conduction.

**Figure 3:**
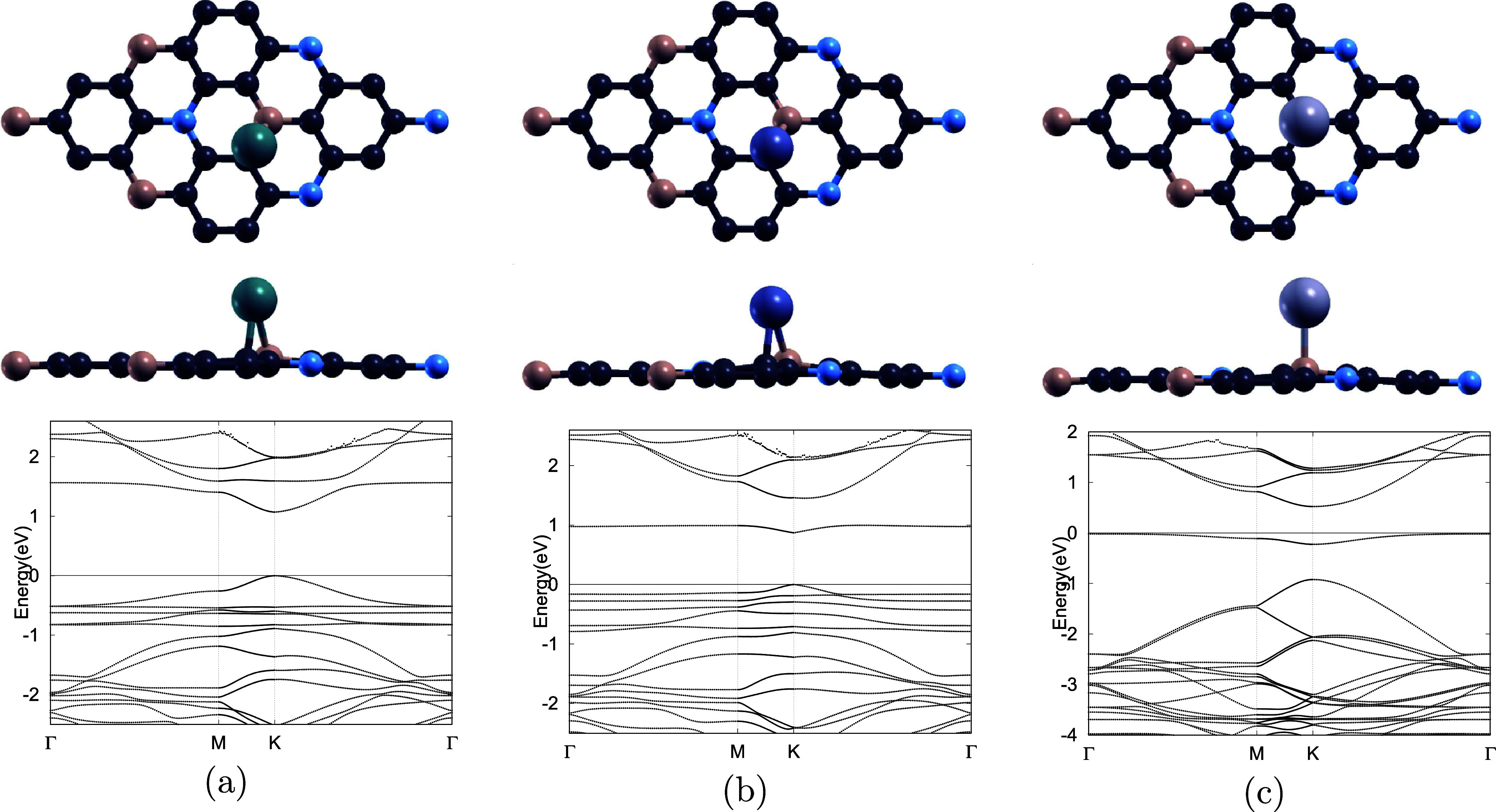
Top and side views along with corresponding band structures of (a) Pd-doped, (b) Pt-doped, and (c) Ag-doped BC_6_N monolayers. For each system, the top, middle, and bottom rows display the top view, side view, and electronic band structure, respectively. B, C, and N atoms are depicted in pink, dark gray, and blue, respectively, while Pd, Pt, and Ag dopants are represented by teal, indigo-gray, and light silver colors. The Fermi level is set to zero for all band structure diagrams, and all structures retain semiconducting behavior after doping.

The optimized structural and electronic properties of the doped monolayers are summarized in Table 3. The positive defect formation energies (*E*_form_) result from the endothermic nature of interstitial doping, where incorporating a foreign atom into the stable 2D lattice requires energy input. Despite being nonspontaneous, these values may be feasible for synthesis via techniques like ion implantation.^88^ However, a full confirmation of thermodynamic stability through phonon dispersion calculations remains an essential requirement for proposing new materials. Future work will necessarily include these calculations to validate the stability of the doped systems, building upon the computational results identified here.

**Table 3:** Comparison of the Calculated, Theoretical, and Experimental Structural Parameters for the Three VOC Molecules and the Pristine BC_6_N Monolayer, including Bond Lengths, Bond Angles, and Lattice Constant (*a*), along with the Associated Percentage Errors

system	parameter	calculated	previous theory	experimental	error (%)
acetone	C–C (Å)	1.22	1.22 ^70^	1.214 ^89^	0.49
	C–O (Å)	1.52	1.51 ^70^	1.520 ^89^	0.00
	*∠*C–C–O (°)	121.7	121.8 ^70^	122 ^89^	0.25
formaldehyde	C–O (Å)	1.22	1.21 ^90^	1.21 ^91^	0.83
	C–H (Å)	1.10	1.12 ^90^	1.11 ^91^	0.90
	*∠*H–C–H (°)	117.1	N/A	117 ^92^	0.09
isoprene	C=C (Å)	1.34	1.35 ^70^	1.34 ^93^	0.00
	C–C (Å)	1.50	1.50 ^70^	1.512 ^93^	0.79
	*∠*C=C–C (°)	121.5	119.7 ^70^	N/A	N/A
pristine BC_6_N	B–C (Å)	1.47	1.47 ^64^	1.48 ^94^	0.68
	C–C (Å)	1.41	1.42 ^64^	1.42 ^94^	0.70
	C–N (Å)	1.45	1.45 ^64^	1.46 ^94^	0.68
	*a* (Å)	4.975	4.99 ^64^	5.01 ^94^	0.70

Subsequently, we evaluated and compared adsorption energy, adsorption distance, band structure, charge transfer, recovery time, and sensitivity to determine the most effective sensing material for the target molecules.

### VOC Adsorption on Pristine BC_6_N

The optimized molecular structures of acetone, formaldehyde, and isoprene are presented in Figure 1. For acetone, the C–C, C–H, and C–O bond lengths are 1.52, 1.10, and 1.22 Å, respectively, with corresponding bond angles of 121.7° for C–C–O, 116.6° for C–C–C, 110.3° for C–C–H, and 109.8° for H–C–H. Formaldehyde exhibits C–O and C–H bond lengths of 1.22 and 1.10 Å, respectively, along with bond angles of 121.5° for O–C–H and 117.1° for H–C–H. In isoprene, the C=C and C–C bonds measure 1.34 and 1.50 Å correspondingly, while the C–H bonds are 1.09 Å. The associated bond angles are 121.5° for C=C–C, 118.8° for C–C–C, 122.1° for C=C–H, and 115.3° for C–C–H. The geometry of the pristine 2 × 2 BC_6_N monolayer was also optimized, as shown in Figure 2a. The resulting lattice constant is measured to be 4.975 Å, and the B–C, C–C, and C–N bond lengths are 1.47, 1.41, and 1.45 Å, respectively. As displayed in Figure 2b, the calculated band gap is 1.273 eV, which expands upon the zero band gap of pristine graphene. These results show excellent agreement with prior theoretical studies and align closely with available experimental data. A detailed comparison of calculated, theoretical, and experimental parameters is provided in Table 3.

Next, the adsorption behavior of acetone, formaldehyde, and isoprene on the pristine BC_6_N monolayer was investigated. To ensure accuracy, a converged vacuum height of 26, 15, and 20 Bohr was applied to the BC_6_N–acetone, – formaldehyde, and – isoprene systems, respectively. Initial molecular placements were selected based on the optimized adsorption geometries reported by Jiang and Luo and Aghaei et al.,^62,68^ and the resulting relaxed configurations are illustrated in Figure 4.

**Figure 4:**
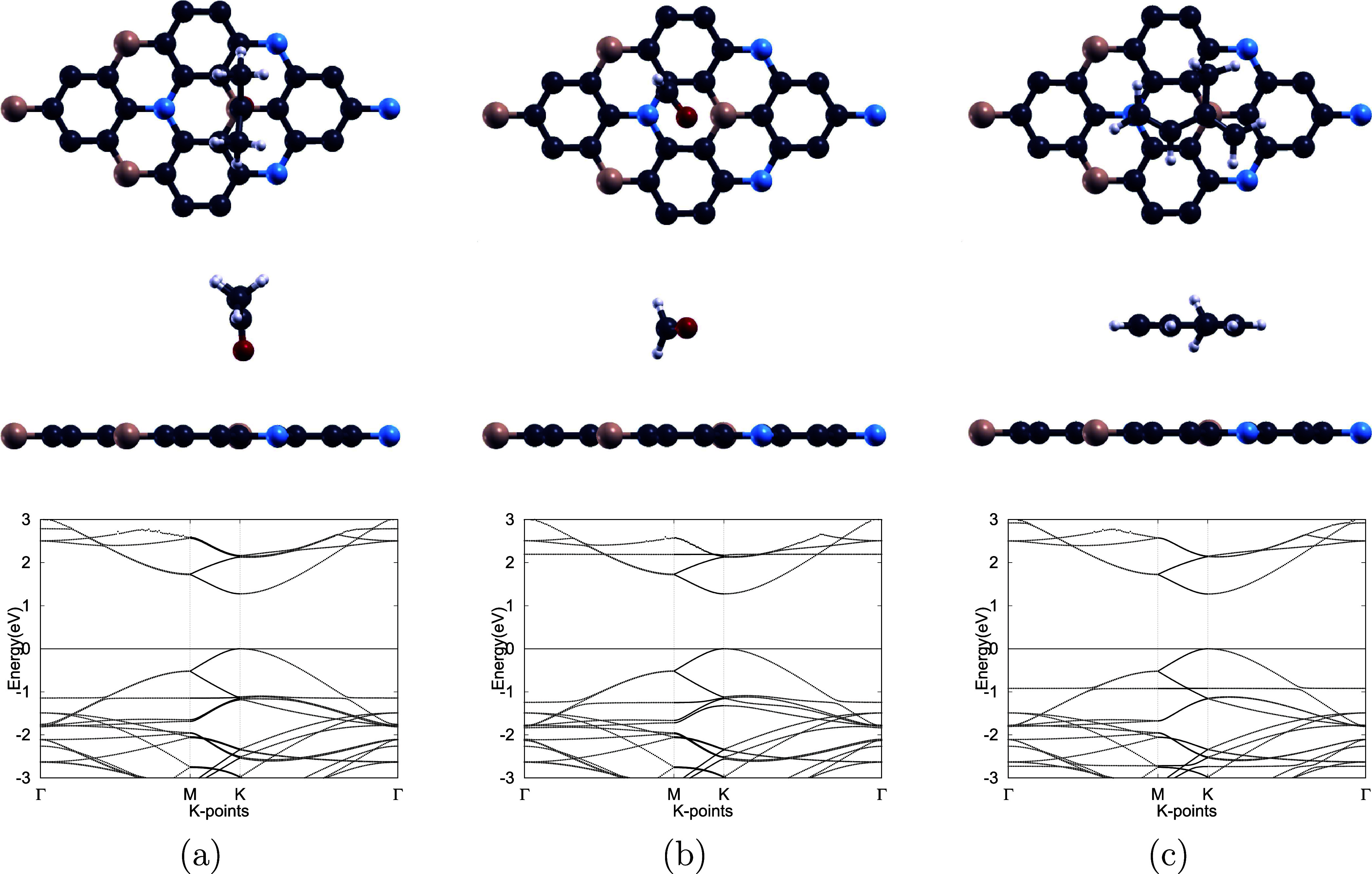
Top and side views of the most stable adsorption geometries (top and middle rows) and corresponding electronic band structures (bottom row) of (a) acetone–, (b) formaldehyde–, and (c) isoprene–BC_6_N systems. H, B, C, N, and O atoms are represented by white, pink, dark gray, blue, and red, respectively. The Fermi level in all band structure diagrams is set to zero.

As shown in Table 4, the minimum adsorption distances for acetone, formaldehyde, and isoprene are found to be 3.27, 4.29, and 4.09 Å, respectively. The atomic radii of B, C, and O atoms are 0.87, 0.67, and 0.48 Å, and the corresponding sums for B–O, C–O, and B–C pairs are 1.35, 1.15, and 1.54 Å, respectively. Since the shortest distances between the VOC molecules and the BC_6_N surface exceed these radii sums, the adsorption is determined to be physical in nature.^64,95,96^

**Table 4:** Adsorption Energy (*E*_ad_), Minimum Adsorption Distance (*d*), Band Gap (*E*_g_), Percentage Change in Band Gap (Δ*E*_g_), and Recovery Time (τ) for VOC Adsorption on the Pristine BC_6_N Monolayer

system	*E*_a_ (eV)	*d* (Å)	*E*_g_ (eV)	Δ*E*_g_ (%)	τ (s)
acetone on BC_6_N	0.001	3.27	1.275	0.19	9.44 × 10^–13^
formaldehyde on BC_6_N	–0.086	4.29	1.274	0.13	2.84 × 10^–11^
isoprene on BC_6_N	–0.013	4.09	1.271	–0.11	1.66 × 10^–12^

This conclusion is further supported by the calculated adsorption energies of 0.0015, −0.0865, and −0.0131 eV for acetone, formaldehyde, and isoprene, respectively. According to prior studies by Muktadir et al. and Bahamon et al., adsorption energies in the range of −0.3 to −0.6 eV are characteristic of physisorption, while values more negative than −0.8 eV typically indicate chemisorption.^97,98^ Thus, the weakly negative adsorption energies for formaldehyde and isoprene indicate low interaction strength and minimal stabilization of the adsorbed complexes, whereas the slightly positive value for acetone suggests nonspontaneous adsorption and an unstable configuration.

In an effective sensing material, adsorption should be not only energetically favorable but also induce a measurable narrowing of the electronic structure.^19^ For BC_6_N, the band gap remains nearly unchanged following VOC adsorption, shifting only from 1.273 eV in the pristine monolayer to 1.275, 1.274, and 1.271 eV for acetone, formaldehyde, and isoprene, respectively. As presented in Figure 4, this negligible variation indicates that VOC adsorption has minimal impact on the monolayer’s electronic properties, correlating with low sensitivity and poor sensing performance.

Recovery time calculations were further performed to assess the reusability of the sensor material. The desorption times for acetone, formaldehyde, and isoprene were estimated to be 9.44 × 10^–13^, 2.84 × 10^–11^, and 1.66 × 10^–12^ s, respectively. In practical gas sensing applications, the adsorption must be sufficiently strong to retain the molecule on the substrate, yet weak enough to enable rapid desorption and sensor recovery. Here, however, the recovery times below one nanosecond are considered impractical for real-time detection, as gas molecules desorb almost immediately, preventing effective signal capture.^19^

Together, these results—summarized in Table 4—highlight the limited suitability of pristine BC_6_N for sensing acetone, formaldehyde, and isoprene due to weak physisorption, long adsorption distances, negligible band gap modulation, and ultrafast recovery times.

### VOC Adsorption on Pd–BC_6_N

To enhance the adsorption performance of pristine BC_6_N, Pd was introduced as an interstitial dopant. As shown in Figure 2a and Table 5, seven potential doping sites were evaluated on the monolayer surface: above the center of the hexagonal ring (*H*), above the B–C, C–C, or C–N bond (*B*), and atop B, C, and N atoms (*T*). Among these, the most energetically favorable site for Pd was found to be above the B–C bond, consistent with prior findings by Jiang and Luo.^62^ Based on eq 1, the corresponding defect formation energy was calculated to be 2.277 eV. After structural optimization, the lattice constant of the monolayer is slightly expanded from 4.975 to 4.981 Å. The Pd–BC_6_N structure also exhibits Pd–B and Pd–C bond lengths of 2.24 and 2.13 Å, respectively and a B–Pd–C bond angle of 39.7°, as shown in Figure 2a. Furthermore, band structure calculations were performed, showing that Pd doping narrows the band gap from 1.273 eV in pristine BC_6_N to 1.069 eV, as listed in Table 2. The reduction in the band gap is a key electronic response of transition metal doping. It arises from two primary mechanisms: (i) the strain or pressure induced by the dopant atoms within the host lattice, which modifies the valence and conduction band widths, and (ii) hybridization between these dopant states and the BC_6_N host states, which shifts the valence band maximum upward and the conduction band minimum downward, thereby directly reducing the fundamental band gap.^99,100^

**Table 5:** Relative Total Energies of Pd-, Pt-, and Ag-Doped BC_6_N Monolayers at the Seven Doping Sites Defined in Figure 2a^a^

doping site	Pd–BC_6_N (eV)	Pt–BC_6_N (eV)	Ag–BC_6_N (eV)
*H*	0.0763	0.2996	0.0000
*B*_B–C_	0.0000	0.0000	0.0019
*B*_C–C_	0.0008	0.0008	0.0071
*B*_C–N_	0.2247	0.3672	0.2417
*T*_B_	0.0843	0.3765	0.0008
*T*_C_	0.0005	0.0605	0.0039
*T*_N_	0.9105	1.3137	0.2436

a For each dopant, the most stable configuration is set to 0.0000 eV, and all other values are reported as energy differences relative to this minimum.

Notably, the Pd–BC_6_N system retains semiconducting properties after doping, as evidenced by its nonzero band gap. It also maintains a direct band gap, and the calculated value is consistent with previously reported results of 1.075 and 1.080 eV.^62,64^

Subsequently, the three VOC molecules were adsorbed onto Pd–BC_6_N, with the optimized configurations shown in Figure 5. Adsorption energies of −0.882, −0.801, and −1.392 eV and minimum adsorption distances of 2.11, 2.07, and 2.13 Å were determined for acetone, formaldehyde, and isoprene, respectively, as displayed in Table 6. Compared to pristine BC_6_N, the shorter adsorption distances and more negative adsorption energies reflect stronger and more spontaneous interactions upon Pd doping. Moreover, the atomic radii of C, O, and Pd are 0.67, 0.48, and 1.69 Å, respectively, corresponding to radii sums of 2.36 Å for Pd–C and 2.17 Å for Pd–O.^101^ As the adsorption distance for each VOC–BC_6_N complex is less than the sum of corresponding atomic radii of the atoms, it can be deduced that all three molecules are chemically adsorbed on the Pd–BC_6_N sheet. This conclusion is further supported by the significant adsorption energies (>0.8 in magnitude), which indicate strong binding interactions.^97,98^ Of the three VOC molecules, isoprene exhibits the strongest adsorption onto the monolayer due to its greatest adsorption energy.

**Table 6:** Adsorption Energy (*E*_ad_), Minimum Adsorption Distance (*d*), Band Gap (*E*_g_), Percentage Change in Band Gap (Δ*E*_g_), and Recovery Time (τ) for VOC Adsorption on the Pd–BC_6_N Monolayer

system	*E*_a_ (eV)	*d* (Å)	*E*_g_ (eV)	Δ*E*_g_ (%)	τ (s)
acetone on Pd–BC_6_N	–0.882	2.11	1.105	3.32	646.98
formaldehyde on Pd–BC_6_N	–0.801	2.07	1.089	1.87	28.08
isoprene on Pd–BC_6_N	–1.392	2.13	1.120	4.75	2.39 × 10^11^

**Figure 5:**
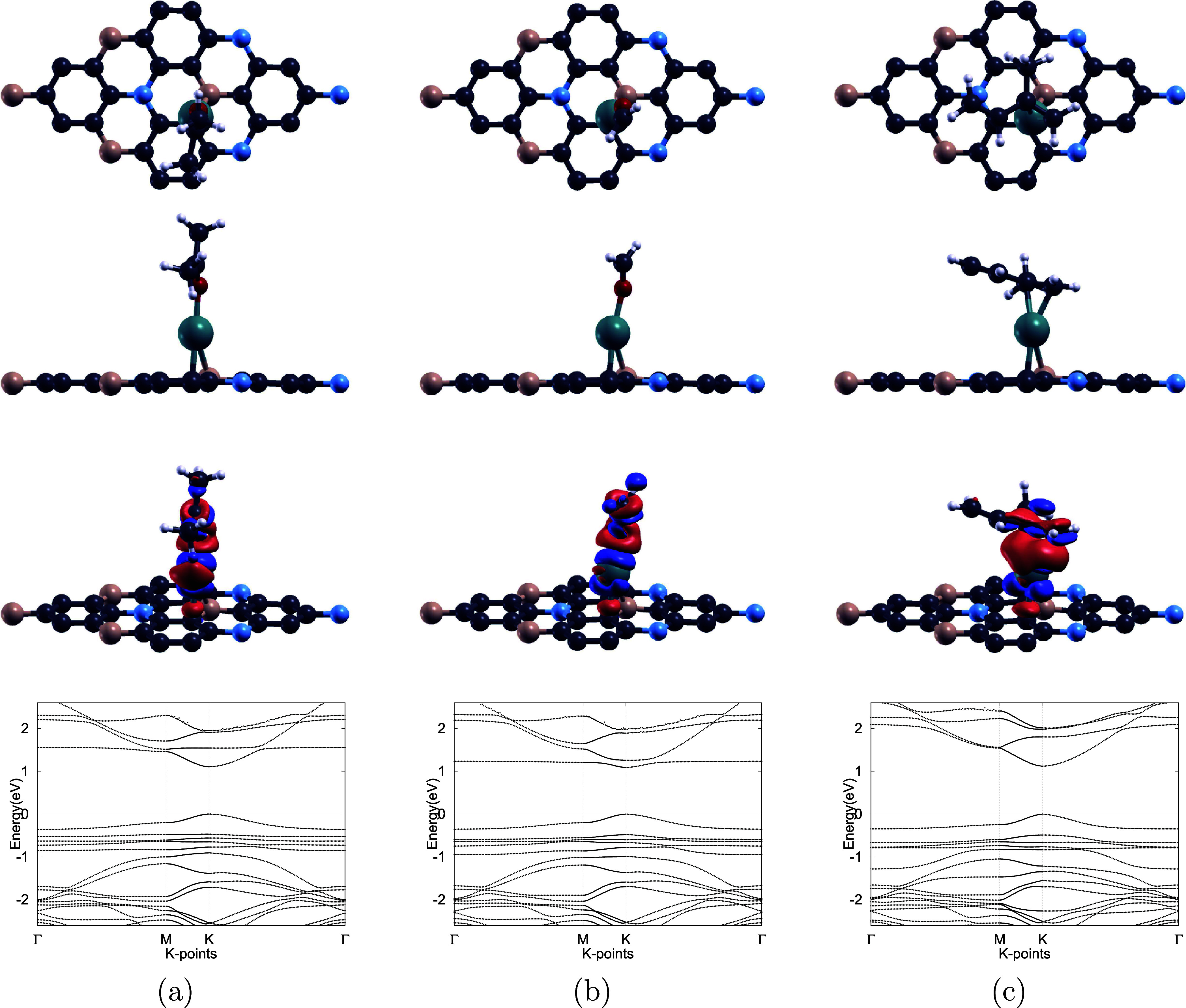
From top to bottom, the four rows present the top views, side views, charge transfer isosurfaces, and electronic band structures of the optimized configurations for (a) acetone, (b) formaldehyde, and (c) isoprene on the Pd–BC_6_N monolayer. In the charge transfer plots, the red and blue colors indicate regions of electron accumulation and depletion, respectively, with an isosurface value of 0.0015 e/Å^3^. The Fermi level is set to zero in all band structure diagrams. H, B, C, N, O, and Pd atoms are shown in white, pink, dark gray, blue, red, and teal, respectively.

Charge transfer analysis provided further insight into the interaction between acetone, formaldehyde, and isoprene and the Pd–BC_6_N monolayer. As shown in Figure 5, the notable overlap between electron accumulation (red) and depletion (blue) regions reflects substantial charge redistribution upon adsorption. Due to their higher electronegativity, the C atoms in isoprene and the O atom in acetone and formaldehyde attract electrons, leading to localized charge accumulation in these regions and depletion around the Pd atom, which acts as an electron donor. These findings, consistent with the significant adsorption energies and shorter adsorption distances, indicate strengthened interactions upon Pd doping.

The electronic band structures of Pd–BC_6_N were computed before and after VOC adsorption, as shown in Figures 2a and 5. The band gap was found to increase from 1.069 eV to 1.105, 1.089, and 1.120 eV upon adsorption of acetone, formaldehyde, and isoprene, corresponding to percentage increases of 3.32, 1.87, and 4.75%, respectively. This change occurs due to the local strain and pressure induced on the Pd–BC_6_N monolayer by the adsorption of the VOC molecules. The dopant and adsorbate introduce compressive stress that modifies the electronic structure by altering the band widths—the energy widths of the valence and conduction bands. This distortion of the band structure consequently shifts the band edge positions, modifying the fundamental band gap.^102^ Although this change is more significant than that observed for pristine BC_6_N, a widening of band gap generally reduces electrical conductivity, thereby weakening the material’s sensing capability.^103^ According to eqs 6 and 7, this expanded band gap results in moderate sensitivities of 49.6, 32.1, and 62.6%, respectively—values that reflect a suboptimal sensing response.

Finally, the recovery time for each VOC–BC_6_N system was calculated to evaluate the reusability of the sensing material. Using eq 5, the estimated recovery times were 646.98 s for acetone, 28.08 s for formaldehyde, and 2.39 × 10^11^ s for isoprene. The relatively shorter recovery time for formaldehyde indicates more facile desorption and favorable reversibility. In contrast, the prolonged desorption times for acetone and isoprene indicate strong binding to the monolayer, thereby limiting the reversibility of Pd–BC_6_N for sensing these VOCs.

Overall, while Pd doping significantly enhances the adsorption strength and electronic interaction with the three molecules, the expanded band gap and long recovery times for certain analytes limit Pd–BC_6_N’s utility as a reusable, high-sensitivity gas sensor.

### VOC Adsorption on Pt–BC_6_N

As with the Pd–BC_6_N monolayer, site selection calculations identified the B–C bond as the most energetically favorable doping site for Pt, as shown in Table 5. The defect formation energy was calculated to be 3.570 eV, indicating a more energy-consuming doping process compared to that of Pd. After structural optimization, the Pt–BC_6_N monolayer exhibits Pt–B and Pt–C bond lengths of 2.20 and 2.06 Å, respectively, with a B–Pt–C bond angle of 41.1°. The lattice constant is also slightly stretched to 4.979 Å, as shown in Table 2. Band structure calculations further reveal a reduced band gap of 0.870 eV compared to pristine and Pd-doped BC_6_N. Similar to Pd–BC_6_N, the Pt-doped monolayer retains semiconducting properties as well as a direct band gap, consistent with prior results reported by Alghamdi et al.^67^

The adsorption behavior of the three VOC molecules on Pt–BC_6_N was subsequently examined. As shown in Table 7, the adsorption energies for acetone, formaldehyde, and isoprene were calculated to be −1.380, −1.292, and −2.211 eV, with corresponding adsorption distances of 2.03, 2.01, and 2.09 Å, respectively. These values indicate stronger and more stable interactions than those observed for Pd–BC_6_N, as reflected by both the greater adsorption energies and shorter bond lengths. To evaluate the nature of adsorption, the observed distances were compared to the sums of atomic radii for Pt–C (2.44 Å) and Pt–O (2.25 Å), based on a Pt atomic radius of 1.77 Å.^101^ Since all adsorption distances are shorter than their corresponding radii sums and the adsorption energies exceed −0.8 eV in magnitude, chemical adsorption can be inferred.^95–98^ Among the three VOCs, isoprene demonstrates the greatest adsorption strength, as reflected by its most negative adsorption energy.

**Table 7:** Adsorption Energy (*E*_ad_), Minimum Adsorption Distance (*d*), Band Gap (*E*_g_), Percentage Change in Band Gap (Δ*E*_g_), and Recovery Time (τ) for VOC Adsorption on the Pt–BC_6_N Monolayer

system	*E*_a_ (eV)	*d* (Å)	*E*_g_ (eV)	Δ*E*_g_ (%)	τ (s)
acetone on Pt–BC_6_N	–1.380	2.03	1.194	37.29	1.54 × 10^11^
formaldehyde on Pt–BC_6_N	–1.291	2.01	1.174	34.98	4.95 × 10^9^
isoprene on Pt–BC_6_N	–2.211	2.09	1.232	41.73	1.42 × 10^25^

Figure 6 shows the charge transfer isosurfaces between the Pt–BC_6_N and the three VOC molecules, computed using eq 4. In all cases, electron depletion occurs around the Pt dopant, while accumulation is observed near the electronegative C and O atoms within the adsorbed molecules. Similar to Pd–BC_6_N, a clear overlap between regions of electron accumulation (red) and depletion (blue) is observed, indicating substantial charge redistribution upon adsorption. Notably, VOC adsorption on Pt–BC_6_N exhibits more pronounced charge transfer than on Pd–BC_6_N, indicating a more significant exchange of electrons and aligning with the larger adsorption energies previously reported.

**Figure 6:**
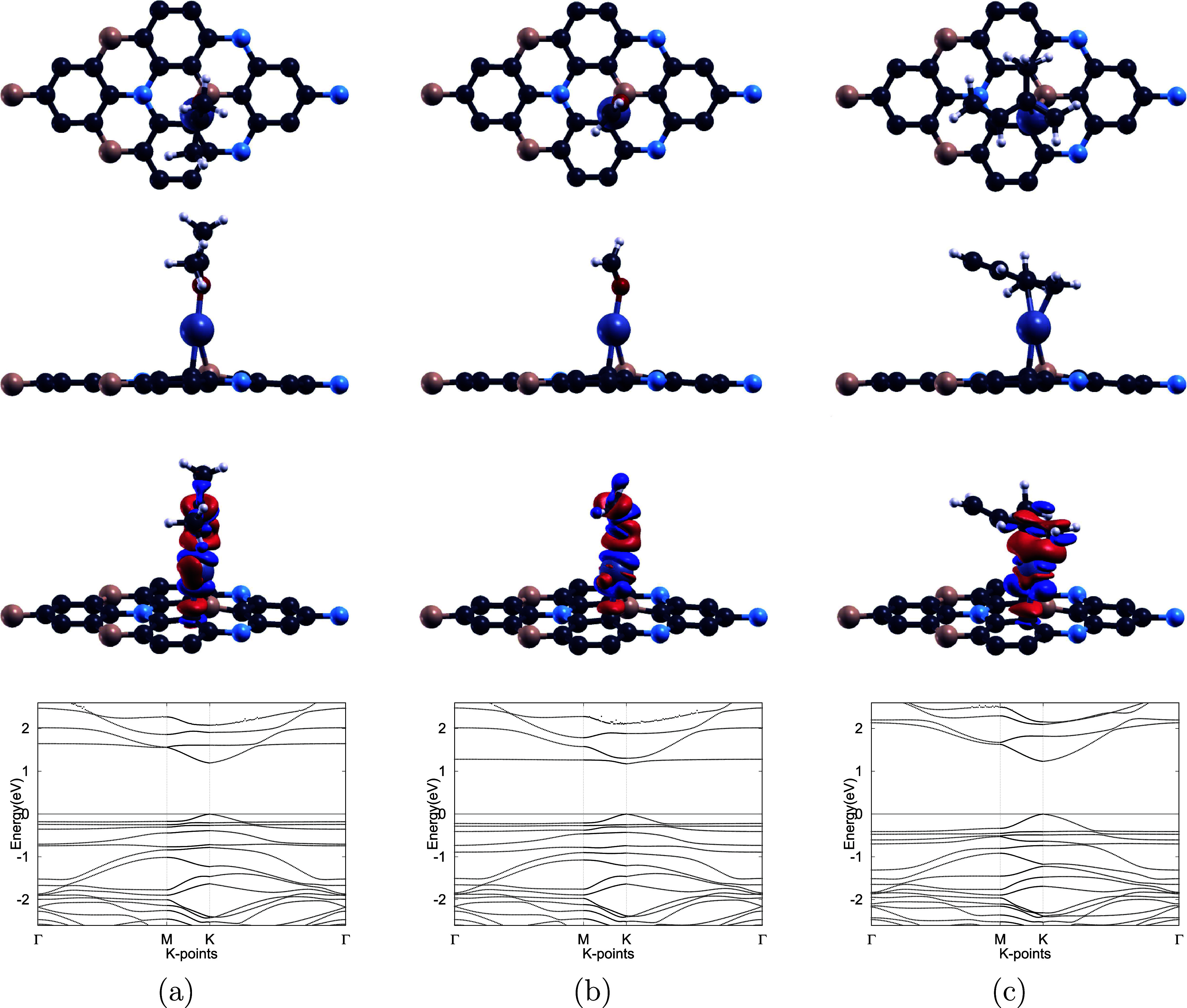
From top to bottom, the four rows show the top views, side views, charge transfer isosurfaces, and band structures of the most stable adsorption configurations for (a) acetone, (b) formaldehyde, and (c) isoprene on the Pt–BC_6_N monolayer. In the charge transfer plots, red and blue regions represent electron accumulation and depletion, respectively, with isosurface values of 0.002 e/Å^3^ for acetone and formaldehyde, and 0.0025 e/Å^3^ for isoprene. The Fermi level is set to zero in all band structure diagrams. H, B, C, N, O, and Pt atoms are shown in white, pink, dark gray, blue, red, and indigo-gray, respectively.

Upon adsorption of acetone, formaldehyde, and isoprene, the electronic band gap of the Pt–BC_6_N monolayer further increases from 0.870 eV to 1.194, 1.174, and 1.232 eV, corresponding to percentage changes of 37.29, 34.98, 41.73%, respectively. This expansion can be attributed to the structural deformation and strain induced on the Pt–BC_6_N surface by the adsorbed molecules, which alters the bandwidth of valence and conduction bands.^102^ These shifts are more substantial than those observed for Pd–BC_6_N, indicating a greater electronic response to VOC adsorption. Reflecting this variation, the calculated sensitivities based on eq 7 are 99.8, 99.7, and 99.9% for acetone, formaldehyde, and isoprene, respectively, indicating that the monolayer exhibits high sensitivity toward all three VOCs.

However, recovery time must also be evaluated to assess the material’s potential for reversible sensing applications. Based on eq 5, the desorption times for acetone, formaldehyde, and isoprene were estimated to be 1.54 × 10^11^, 4.95 × 10^9^, and 1.42 × 10^25^ s, respectively. These exceedingly long times suggest irreversible adsorption and severely limit sensor reusability.

For a practical recovery-type sensor to operate under these conditions, external energy input would be required to overcome the high energy barrier and force desorption. In real-world applications, this is commonly achieved through integrated microheaters that briefly raise the sensor’s temperature (thermal recovery) or through UV light illumination (photoassisted recovery) to provide the necessary activation energy.^104,105^

However, the magnitude of the adsorption energies suggests the required energy input would be significant, potentially impacting power consumption and device longevity. Therefore, while a reusable sensor is theoretically possible with such active recovery systems, the operational drawbacks may be substantial. This critical finding suggests that Pt–BC6N’s optimal application may not be as a reversible sensor, but rather in applications such as a highly sensitive single-use dosimeter for cumulative exposure monitoring, where irreversibility is a key asset.^106^

### VOC Adsorption on Ag–BC_6_N

Given the poor recovery times observed for Pd– and Pt–BC_6_N, Ag was selected as a dopant to assess its potential in enhancing sensor reusability. Previous studies by Zhu and Luo and Tan et al. have shown that Ag-doped graphene and SnS_2_ exhibit favorable adsorption strength coupled with rapid desorption of gas molecules, prompting a similar evaluation of Ag–BC_6_N.^19,107^ As shown in Table 5, the most energy-stable doping site for Ag is identified to be above the B atom, yielding a defect formation energy of 2.436 eV. The optimized configuration, illustrated in Figure 3c, indicate a nearly unchanged lattice constant of 4.976 Å and an Ag–B bond length of 2.38 Å. Band structure analysis reveals a band gap of 0.543 eV—the narrowest among the doped systems studied. Unlike Pd– and Pt–BC_6_N, Ag–BC_6_N retains semiconducting properties yet exhibits an indirect band gap after doping, as displayed in Figure 3, reflecting a distinct modification of the electronic structure.

VOC adsorption on Ag–BC_6_N was then examined. Figure 7 shows the optimized structures of the VOC–BC_6_N systems, with the adsorption energies calculated to be −0.297, −0.295, and −0.535 eV for acetone, formaldehyde, and isoprene, respectively. This corresponds with adsorption distances of 2.31, 2.24, and 2.23 Å, respectively. Compared to Pd– and Pt–BC_6_N, Ag doping results in weaker interactions, as evidenced by the less negative adsorption energies and slightly longer adsorption distances. Given the atomic radius of Ag (1.65 Å), the Ag–C and Ag–O radius sums are 2.32 and 2.13 Å, respectively. The observed distances, being close to or slightly below these sums, suggest physisorption or borderline chemisorption. This is further supported by the adsorption energies, which fall within the typical physisorption range (−0.3 to −0.6 eV). As with the other doped systems, isoprene exhibits the strongest interaction among the three VOCs.

**Figure 7:**
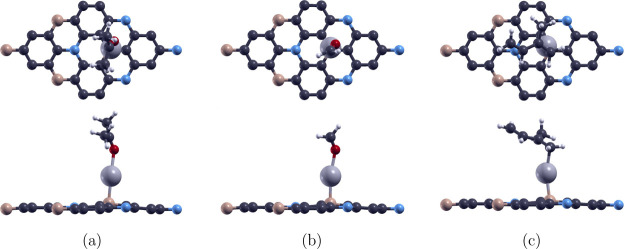
Top and side views of the optimized configurations of (a) acetone, (b) formaldehyde, and (c) isoprene adsorption on the Ag–BC_6_N monolayer. The white, pink, dark gray, blue, red, and light silver colors represent H, B, C, N, O, and Ag atoms, respectively.

We subsequently computed the charge transfers for the Ag–BC_6_N systems, shown in Figure 8. Calculated based on eq 4, a noticeable overlap between red and blue is observed, indicating the exchange of electrons upon adsorption. As with Pd– and Pt–BC_6_N, electrons tend to locally accumulate around the C and O atoms of the VOCs while depleting near the Ag dopant. However, the extent of charge transfer appears less pronounced than in the Pd- and Pt-doped systems, aligning with the less negative adsorption energies and longer interaction distances.

**Figure 8:**
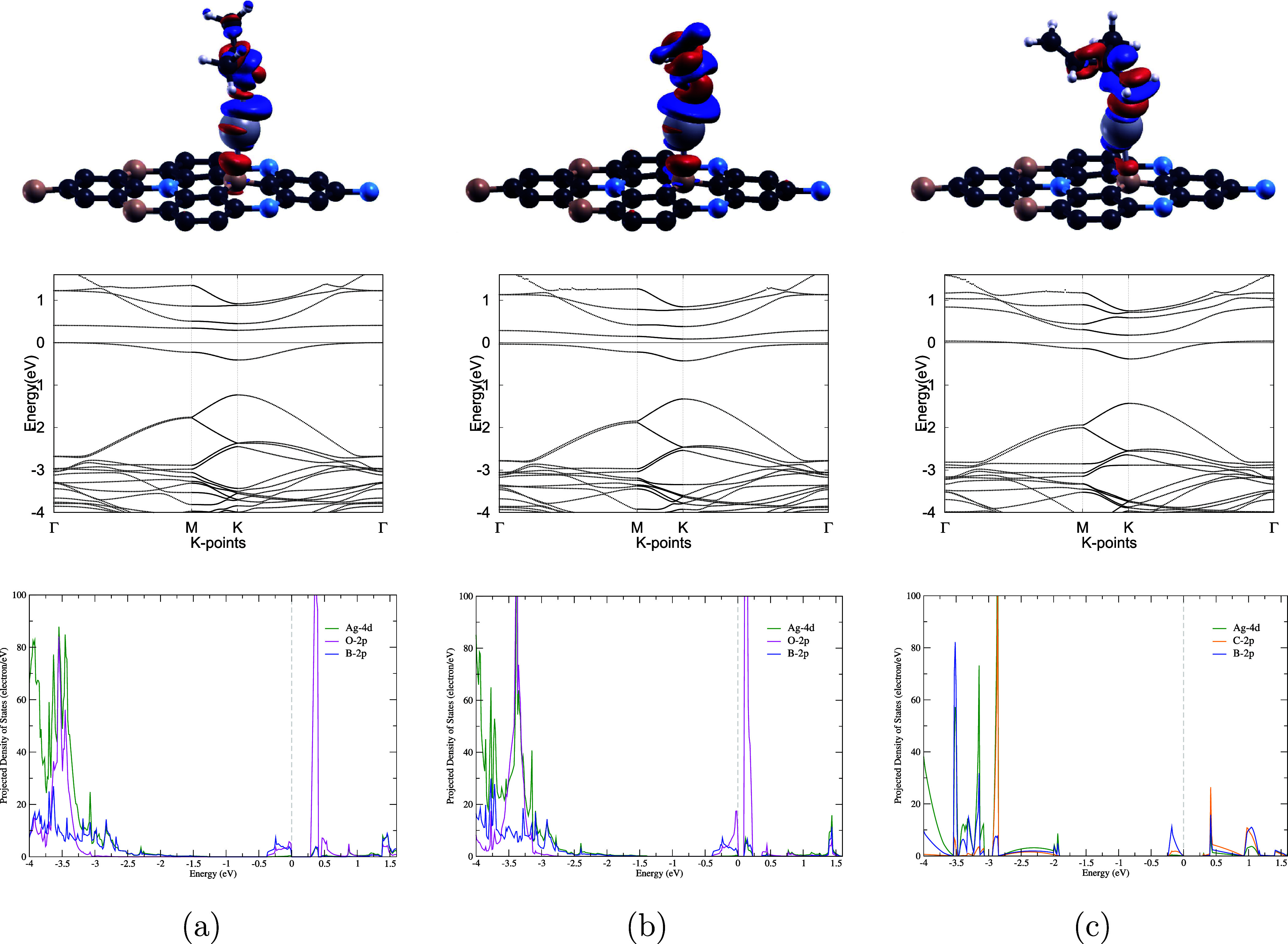
Charge transfer (top row), electronic band structures (middle row), and PDOS (bottom row) of (a) acetone, (b) formaldehyde, and (c) isoprene on a Ag–BC_6_N monolayer. For the charge transfer plots, regions of electron accumulation and depletion are shown in red and blue colors, respectively. The isosurface values are set to 0.001 e/Å^3^ for acetone and formaldehyde, and 0.0015 e/Å^3^ for isoprene. H, B, C, N, O, and Ag atoms are depicted in white, pink, dark gray, blue, red, and light silver, respectively. All VOC-adsorbed complexes maintain semiconducting behavior. The Fermi level is set to zero in the band structure diagrams. Meanwhile, the Ag 4d, O 2p, C 2p, and B 2p orbitals in the PDOS diagrams are represented by green, magenta, orange, and blue colors, respectively.

Figures 3 and 8 present the band structure of Ag–BC_6_N before and after VOC adsorption, respectively. In contrast to the band gap expansion observed for the Pd– and Pt–BC_6_N systems, Ag doping leads to a narrowing of the band gap from 0.543 eV to 0.291, 0.120, and 0.139 eV upon adsorption of acetone, formaldehyde, and isoprene, respectively. This corresponds with substantial percentage decreases of −46.27, −77.88, and −74.35%, as listed in Table 8. This reduction occurs due to the compressive pressure and strain exerted on the Ag–BC_6_N monolayer upon VOC adsorption.^102^ As shown in Figure 8, the Ag–BC_6_N monolayer exhibits semiconducting behavior after the adsorption of all three VOCs, with no transition to metallic character.

**Table 8:** Adsorption Energy (*E*_ad_), Minimum Adsorption Distance (*d*), Band Gap (*E*_g_), Percentage Change in Band Gap (Δ*E*_g_), and Recovery Time (τ) for VOC Adsorption on the Ag–BC_6_N Monolayer

system	*E*_a_ (eV)	*d* (Å)	*E*_g_ (eV)	Δ*E*_g_ (%)	τ (s)
acetone on Ag–BC_6_N	–0.297	2.31	0.291	–46.27	9.84 × 10^–8^
formaldehyde on Ag–BC_6_N	–0.295	2.24	0.120	–77.88	9.03 × 10^–8^
isoprene on Ag–BC_6_N	–0.535	2.23	0.139	–74.35	9.74 × 10^–4^

Previous theoretical studies by Ansari et al. and Suriya et al. have shown that a decrease in band gap upon gas adsorption typically enhances electrical conductivity, thereby improving the sensor’s responsiveness.^103,108^ Based on eq 7, the corresponding sensitivities for the Ag–BC_6_N systems exceed 200% for all three VOCs, underscoring the material’s ultrahigh sensing response and strong potential for trace-level gas detection. This result can be justified from the exponential relationship between electrical conductivity and band gap, as per eqs 6 and 7. While the isosurface values for acetone, formaldehyde, and isoprene adsorption indicate a smaller magnitude of charge transfer compared to the Pd– and Pt–BC_6_N systems, the critical factor for sensitivity is not the charge redistribution, but its effect on the band gap. In Ag–BC_6_N, a significant band gap reduction is induced, which in turn triggers an exponential increase in conductivity, resulting in high sensitivity.

Furthermore, the recovery times for acetone, formaldehyde, and isoprene adsorption on Ag–BC_6_N were calculated to be 9.84 × 10^–8^, 9.03 × 10^–8^, and 9.74 × 10^–4^ s, respectively, as listed in Table 8. These values reflect an exceptionally rapid desorption process and indicate strong reversibility, supporting the potential of Ag–BC_6_N for reusable sensing. Among the three VOCs, isoprene demonstrates the most promising behavior, with a recovery time appropriately exceeding one nanosecond—sufficient to enable stable adsorption while ensuring timely desorption under practical conditions. This combination of swift desorption kinetics, appreciable adsorption energies, and favorable band gap modulation underscores Ag–BC_6_N’s viability as a reusable gas sensor, surpassing Pd– and Pt–BC_6_N in practical sensing performance.

Given Ag–BC_6_N’s strong promise in VOC sensing, the projected density of states (PDOS) was also computed to further examine the electronic interactions of the three VOCs with the doped monolayer, as shown in Figure 8. For acetone adsorption, electron hybridization between Ag 4d, O 2p, and B 2p orbitals occur at approximately −3.9, −3.7, −3.6, and 0.4 eV. Similarly, the overlapping peaks at around −3.8, −3.7, −3.5, and 0.1 eV between Ag 4d, O 2p, and B 2p were observed for the BC_6_N–formaldehyde system. For isoprene, the PDOS diagram reveals hybridization between Ag 4d, C 2p, and B 2p orbitals at approximately −3.5, −3.1, −2.7, and 0.4 eV. The overlapping peaks near the Fermi level, particularly those at or near 0.1 eV, indicate the formation of new hybridized states that are responsible for the band gap modulation and heightened conductivity after adsorption. This strong electronic coupling—primarily between the Ag 4d and the O 2p orbitals of formaldehyde—directly influences the high adsorption energy and charge transfer. Therefore, these PDOS results provide direct electronic structure evidence that explains the strong adsorption and the resulting high sensing response.

For more insight on Ag–BC_6_N’s performance, a comparison with existing literature is invaluable. Kumar et al. investigated the adsorption of various pancreatic cancer biomarkers, including 2-pentanone and 4-ethyl-1–2-dime-thylbenzene, on pure and TM-doped Ti_3_C_2_T_*x*_ MXenes. Adsorption energies were found within the range −0.60 to −1.10 eV for the pristine MXenes, while adsorption distances generally fell between 2.10 to 2.50 Å.^48^ This is comparative to many parameters determined within this study, though the more negative adsorption energies may indicate stronger adsorption interaction compared to Ag–BC_6_N. Panigrahi et al. similarly investigated the adsorption behavior of lung cancer biomarkers, including isoprene, on Ti_3_C_2_T_*x*_ MXenes, revealing a range of adsorption energies from −0.505 to −3.49 eV for the molecule.^47^ This too indicates a substantially stronger adsorption strength, similar to the ranges achieved for Pd– and Pt–BC_6_N. Comparatively, other materials of interest, including MoS_2_ and MoSi_2_N_4_ monolayers, have also been investigated for the detection of lung cancer, as shown in studies by Panigrahi et al. and Alfalasi et al. Ag–BC_6_N indicates recovery times, charge transfer, and orbital hybridization on par with results from these studies, while parameters such as sensing response may even improve upon determined ranges of 16.2 to 26.6%.^109,110^

The practical deployment of a breath sensor also requires consideration of its selectivity against common interfering gases present in exhaled breath, including H_2_O, CO_2_, NH_3_, and ethanol.^111^ While a computational screening of all potential interferents was not undertaken here, information can be drawn from literature on similar 2D materials, where properties such as adsorption energy, electronic response, and sensitivity contribute to differentiating between relevant gases.^112–114^

Crucially, prior theoretical studies on BC_6_N systems provide strong evidence of selectivity against many common interferents. Aasi et al. demonstrated that both pristine and Pd-doped BC_6_N exhibit low sensitivity (<10%) toward CO, CO_2_, NH_3_, H_2_O, H_2_S, and SO_2_, as evidenced by weak physisorption and minimal electronic response.^64^ This is corroborated by a study by Yu et al., who reported that pristine BC_6_N shows weak interactions with CO_2_, characterized by small adsorption energies and long bond distances.^115^

However, a study by Aghaei et al. indicates the material’s high sensitivity and adsorption strength toward ethanol, specifically after the incorporation of a single carbon vacancy.^68^ This suggests that while TM-doped BC_6_N is likely effective at distinguishing target VOCs from smaller, inert molecules like CO_2_ and H_2_O, achieving selectivity against VOCs with similar chemical properties—such as ethanol—remains a challenge and requires further experimental validation.

Therefore, for practical deployment, we propose integrating the TM-doped nanosensor with a selective prefilter, such as a molecular imprint,^116^ separation column,^117^ or membrane.^117^ This integrated system would preprocess the breath sample to remove bulk interferents, thereby leveraging the high sensitivity of the Ag–BC_6_N monolayer for the detection and quantification of the target biomarkers within a practical diagnostic device^118^

## Conclusions

In this study, we used first-principles calculations based on DFT to investigate the adsorption and electronic properties of acetone, formaldehyde, and isoprene on pristine and transition-metal-doped BC_6_N monolayers. Based on our analysis of adsorption energy, adsorption distance, band structure, charge transfer, PDOS, and sensitivity, the following conclusions can be drawn:

1. Pristine BC_6_N shows limited sensing capability toward the selected VOCs, as indicated by weak adsorption energies, long binding distances, minimal band gap modulation, and low sensitivity.
2. Pd– and Pt–BC_6_N exhibit strong chemical adsorption, reflected by large adsorption energies, short bond lengths, and significant charge transfer. However, these systems are limited by band gap widening upon adsorption and extremely long recovery times, reducing their sensitivity and reusability.
3. Ag–BC_6_N improves upon Pd and Pt doping by showing moderate adsorption strength, high sensitivity, and short recovery times. These features suggest excellent reversibility and position Ag–BC_6_N as a promising candidate for practical gas-sensing applications.

Future work should evaluate the material’s selectivity, operational stability, and energy efficiency under realistic environmental conditions to further assess its potential in noninvasive biomedical sensing platforms. Incorporating van der Waals corrections and performing phonon dispersion calculations will also be essential to improve the quantitative accuracy of adsorption properties and confirm the thermodynamic stability of the doped systems.
